# Slowly but surely: gradual diversification and phenotypic evolution in the hyper-diverse tree fern family Cyatheaceae

**DOI:** 10.1093/aob/mcz145

**Published:** 2019-09-28

**Authors:** Oriane Loiseau, Anna Weigand, Sarah Noben, Jonathan Rolland, Daniele Silvestro, Michael Kessler, Marcus Lehnert, Nicolas Salamin

**Affiliations:** 1 Department of Computational Biology, University of Lausanne, 1015 Lausanne, Switzerland; 2 Institute for Systematic and Evolutionary Botany, University of Zurich, 8008 Zurich, Switzerland; 3 Nees Institute for Biodiversity of Plants, Rheinische Friedrich-Wilhelms University Bonn, Bonn, Germany; 4 Department of Zoology, University of British Columbia, #4200-6270 University Blvd, Vancouver, B.C., Canada; 5 Department of Biological and Environmental Sciences, University of Gothenburg, Gothenburg, Sweden; 6 Global Gothenburg Biodiversity Center, Gothenburg, Sweden; 7 Department of Geobotany and Botanical Garden, Herbarium, Martin-Luther-University Halle-Wittenberg, Neuwerk 21, 06108 Halle, Germany

**Keywords:** Diversification, macroevolution, tree ferns, Cyatheaceae, phylogeny, target sequencing, climatic preferences, fossilized birth–death, phenotypic evolution, gradual evolution, species richness, divergence times

## Abstract

**Background and Aims:**

The tremendously unbalanced distribution of species richness across clades in the tree of life is often interpreted as the result of variation in the rates of diversification, which may themselves respond to trait evolution. Even though this is likely a widespread pattern, not all diverse groups of organisms exhibit heterogeneity in their dynamics of diversification. Testing and characterizing the processes driving the evolution of clades with steady rates of diversification over long periods of time are of importance in order to have a full understanding of the build-up of biodiversity through time.

**Methods:**

We studied the macroevolutionary history of the species-rich tree fern family Cyatheaceae and inferred a time-calibrated phylogeny of the family including extinct and extant species using the recently developed fossilized birth–death method. We tested whether the high diversity of Cyatheaceae is the result of episodes of rapid diversification associated with phenotypic and ecological differentiation or driven by stable but low rates of diversification. We compared the rates of diversification across clades, modelled the evolution of body size and climatic preferences and tested for trait-dependent diversification.

**Key Results:**

This ancient group diversified at a low and constant rate during its long evolutionary history. Morphological and climatic niche evolution were found to be overall highly conserved, although we detected several shifts in the rates of evolution of climatic preferences, linked to changes in elevation. The diversification of the family occurred gradually, within limited phenotypic and ecological boundaries, and yet resulted in a remarkable species richness.

**Conclusions:**

Our study indicates that Cyatheaceae is a diverse clade which slowly accumulated morphological, ecological and taxonomic diversity over a long evolutionary period and provides a compelling example of the tropics as a museum of biodiversity.

## INTRODUCTION

The distribution of biodiversity is strikingly unbalanced when comparing species richness among lineages and one of the main challenges in evolutionary biology is to understand the underlying factors that are responsible for this uneven distribution (Wiens, 2017). This challenge is commonly tackled by comparing the patterns of diversification among regions and taxonomic groups (Antonelli *et al.*, 2015; Eiserhardt *et al.*, 2017), but this requires comparing large numbers of species to have a good understanding of the potential processes involved. Fortunately, the increasing availability of DNA sequence data coupled with advances in phylogenetic comparative methods over recent decades provides us with powerful tools to study the assembly of Earth’s biodiversity through time and space.

The heterogeneity of species richness across clades is likely the result of variations in the rates of speciation or extinction (Wiens, 2017). Changes in diversification rates, measured as the difference between the estimated rates of speciation and extinction, have been attributed to a wide range of factors. Intrinsic traits such as the tank habit in bromeliads (Silvestro *et al.*, 2014) or heterostyly in primroses (de Vos *et al.*, 2014) have been linked with accelerated diversification. Ecological interactions, for instance clownfish mutualism with sea anemones (Litsios *et al.*, 2012) or plant interactions with pollinators (Breitkopf *et al.*, 2015; Serrano-Serrano *et al.*, 2017), have also been hypothesized to play a role in species diversification. Finally, the ecological niche can also affect the diversity between lineages, as shown in the large fern family Polypodiaceae, where lineage diversification is positively associated with the colonization of a wider elevation range (Sundue *et al.*, 2015).

Clade age has also been proposed as an explanation for differences in species richness between groups of organisms through the ‘clade-age’ hypothesis, which argues that older clades tend to have more species simply because they have had more time to accumulate diversity (Wiens, 2017). In this case, species richness results from the steady accumulation of lineages through time rather than from shifts in the rates of diversification. Stable evolutionary dynamics have been reported, for example, in Neotropical Troidini butterflies (Condamine *et al.*, 2012) and several plant groups, such as figs (Bruun-Lund *et al.*, 2018), malagasy *Angraecum* orchids (Andriananjamanantsoa *et al.*, 2016), Annonaceae (Couvreur *et al*., 2011b), Australian Proteaceae (Cardillo and Pratt, 2013) and liverworts (Wilson *et al.*, 2007). The clade-age hypothesis also provides a possible scenario to explain geographical variation in species richness, whereby more diverse regions, for instance the tropics, would be the result of early colonizations rather than variation in the rate of clade diversification among regions (Brown, 2014). In contrast to the clade-age hypothesis, a recent study has proposed that diversification is a time-dependent process whereby younger clades diversify more rapidly than older clades (Henao Diaz *et al.*, 2019). This time dependency could potentially disrupt a correlation between the age of a clade and its diversity.

Alternatively, instead of invoking a single underlying factor, some studies have proposed a more nuanced model of evolutionary dynamics where both time and rates of diversification contribute to the explanation of diversity patterns. For instance, the palm family (Arecaceae) exhibited a constant rate of diversification during most of its long evolutionary history, but several increases in the rate of diversification have punctuated the recent evolution of this group (Couvreur *et al*., 2011a; Baker and Couvreur, 2013). Similarly, the assembly of fern diversity appears to have been shaped by a strong turnover of clades resulting from the combination of both diversity-dependent speciation and climate-driven extinction (Lehtonen *et al.*, 2017).

In this context, the long-term drivers of the diversification history of old and species-rich plant clades can give important insights into the build-up of biodiversity through time. We focused here on the scaly tree fern family Cyatheaceae, an iconic plant group of humid forests comprising about 660 known species distributed worldwide in tropical and subtropical regions. This species richness is remarkable compared with the seven other families of the Cyatheales, which contain from a single species in the Thyrsopteridaceae to ~35 species in the Dicksoniaceae (PPG, 2016). Phylogenetic studies have estimated the divergence between the Cyatheaceae and its sister family the Dicksoniaceae around 145 Ma and have shown that both Gondwanan vicariance and long-distance dispersal contributed to generating the current distribution of scaly tree ferns (Korall and Pryer, 2014). Significant among-clade variations in the rate of diversification have been reported for the family (Janssen *et al.*, 2008; Ramírez-Barahona *et al.*, 2016), and a recent study also found a positive association between the rates of morphological and climatic niche evolution and the rates of diversification, suggesting that the evolutionary dynamics of Cyatheaceae followed those of an adaptive radiation (Ramírez-Barahona *et al.*, 2016). However, only about 10 % of the total number of Cyatheaceae species were represented in these studies. Such a low taxonomic sampling weakens phylogenetic comparative analyses (Heath *et al.*, 2008; Silvestro *et al.*, 2011) and calls into question our understanding of the evolutionary history of the group.

Here, we combined molecular data at an unprecedented level of taxonomic sampling with fossil information to reconstruct a new dated phylogenetic tree of the Cyatheaceae. The origin of the group dates back to the Jurassic and recent theory suggests that diversification in such an old group could be the result of a steady but slow accumulation of species through time (Henao Diaz *et al.*, 2019). Alternatively, the high diversity of the family could be associated with the evolution of intrinsic or ecological traits in specific lineages driving species diversification (Ramírez-Barahona *et al.*, 2016). We used macroevolutionary models to test several hypotheses related to species and trait diversification of the Cyatheaceae. We first tested whether the build-up of species diversity in this group followed the expectation of the clade-age hypothesis, which would imply that species richness in this family increased at a steady rate through its long evolutionary history. As an alternative hypothesis, we tested instead whether their great diversity originated from variations in the rate of diversification between lineages. Second, we investigated the role played by morphological and ecological traits during the evolution of the group by reconstructing the evolutionary dynamics of these traits through time and testing their impact on the rates of diversification. Our findings show that, unlike many species-rich groups, the family Cyatheaceae is an old lineage that diversified at an exceptionally low and homogeneous pace without undergoing extensive morphological evolution. Although the rate of evolution of climatic preferences accelerated in several clades, likely in response to dispersals to higher elevation habitats, this was decoupled from the diversification of the group.

## MATERIALS AND METHODS

### Taxonomic sampling

Our dataset includes 323 species of Cyatheaceae representing ~49 % of the total extant diversity of the group. All genera were included, with sampling fractions of 49 % in *Alsophila*, 47 % in *Cyathea*, 72 % in *Gymnosphaera* and 49 % in *Sphaeropteris*. Sixty-six accessions were previously published and we took them directly from GenBank. The rest of the sampled material (257) was collected and identified personally (by one of the authors); voucher material for published sequences (Korall et al., 2007) was also revised (by one of the authors). For taxonomy we follow the recent reinstatement of *Gymnosphaera* as a separate genus from *Alsophila* (Dong and Zuo, 2018). As outgroup, we included 12 species from three genera (*Calochlaena*, *Dicksonia* and *Lophosoria*) of the sister family Dicksoniaceae.

### DNA extraction, amplification and sequencing

DNA extraction was performed with the NucleoSpin^®^ Plant II (Macherey Nagel, Düren, Germany) kit following the manufacturer’s protocol. We generated sequence data for three chloroplast markers: *trnL*-*trnF* [including the *trnL* group I intron (g1i) and the *trnL*-*trnF* intergenic spacer (IGS)], *trnG*-*trnR* [including the *trnG* group II intron (g2i) and the *trnG*-*trnR* IGS) and *rpl16* (*rps3*-*rpl16*, including parts of the *rps3* exon, as well as the *rps3*-*rpl16* IGS and the *rpl16* g2i]. For PCR and sequencing reactions of *trnG-R*, the primer set and PCR program described by Nagalingum *et al.* (2007) were used. The PCR and sequencing reactions of *trnL-F* and *rpl16* followed the protocol by Noben *et al.* (2017). Reactions were performed either on a Biometra TProfessional TRIO thermocycler (Biometra, Göttingen, Germany) or on an Eppendorf Mastercycler EPGradient S (Eppendorf, Hamburg, Germany). For sequencing, the services of Macrogen Europe (Amsterdam, Netherlands) and GATC Biotech (Konstanz, Germany) were used. All the sequences newly generated in this study are deposited in GenBank (GenBank numbers will be added upon acceptance).

### Phylogenetic analyses

All sequences were aligned using the MAFFT (Katoh *et al.*, 2002) plug-in in Geneious 6.1.8 (Biomatters). Alignments were visually inspected and ambiguous regions were excluded. The three markers were concatenated into a single alignment using SequenceMatrix 1.7.8 (Vaidya *et al.*, 2011). We partitioned the alignment by genes and the best model of nucleotide substitution for each region was selected based on the Akaike information criterion using jModelTest 2.1.7 (Darriba *et al.*, 2012). Although additional chloroplast genes are available with relatively good species sampling in GenBank, we did not use these sequences for our final analyses since they increased the proportion of missing data and adding them to our own molecular dataset resulted in a similar topology (see Supplementary Data for details), similar branch length estimates (Supplementary Data Fig. S1) and did not improve substantially the resolution of the estimated phylogenetic tree (Supplementary Data Figs S2 and S3). Phylogenetic inference and divergence time estimates were made using the fossilized birth–death (FBD) model (Heath *et al.*, 2014) implemented in BEAST 2.4.7 (Bouckaert *et al.*, 2014). Unlike traditional node calibration, FBD analysis allows the use of all available fossil evidence and not only the oldest unequivocal fossil for a given clade. Hence, we selected a set of 41 fossils of Cyatheales, 13 of which belonged to the Cyatheaceae, and constrained their placement using taxonomic information (Supplementary Data Table S1). Importantly, we differ from previous phylogenetic studies of tree ferns regarding the placement of the fossil *Kuylisporites mirabilis* (93.9–100.5 Myr; Mohr and Lazarus, 1994), which has been used to calibrate the crown node of Cyatheaceae based on its resemblance to spores of extant species of *Cyathea* and *Alsophila*. However, this interpretation was due to a misleading taxonomic treatment (the species *Cyathea decurrens* used to be treated as *Alsophila decurrens*) and the *Kuylisporites* spore type is actually only found in extant species of *Cyathea* and not in any other genus. Therefore, in our FBD analysis this fossil was only allowed to be placed in the *Cyathea* lineage and we used the age intervals of the fossils to indicate their respective age. The first appearance of the fossil genus *Cyathocaulis* (Upper Jurassic, 145–165.5 Ma) was used to constrained the origin of the Cyatheaceae (see Supplementary Data for details; Korall et al., 2014). We performed two runs of 500 million Markov chain Monte Carlo (MCMC) generations in BEAST 2.4.7, sampling every 50 000 generations. Convergence and effective sample size (ESS) were checked in Tracer 1.5.0 (Rambaut and Drummond, 2009). The two posterior distributions of trees were combined after removal of a burn-in of 10 % using LogCombiner. We used TreeAnnotator to obtain the maximum clade credibility (mcc) tree after removing the fossil taxa from the combined posterior distributions of trees. In all subsequent macroevolutionary analyses, outgroup species were removed from the phylogenetic tree. Additionally, for comparison we performed divergence time estimation using traditional node dating using four fossil calibrations, including *K. mirabilis* to calibrate the stem node of *Cyathea* (see Supplementary Data for details). We also assessed the effect of using single versus multiple occurrences for each fossil during the FBD analyses (Supplementary Data Figs S5 and S6).

### Diversification rate analyses

We used a variety of approaches to estimate net diversification rates across the Cyatheaceae family and to assess the presence of rate heterogeneity. First, we used BAMM 2.5 (Rabosky, 2014) to infer lineage-specific diversification rates on the mcc tree from BEAST. We specified clade-specific sampling fractions to account for non-random incomplete taxon sampling. Given the recent debate on prior sensitivity of the BAMM model (Moore *et al.*, 2016; Rabosky *et al.*, 2017), we ran the analysis under different priors for the number of expected shifts (expectedNumberofShifts = 0.1; 1; 5; 10; 50) to assess the robustness of our result to prior parameterization. Each analysis was run for 20 million generations, sampling every 2000 generations. Convergence and ESS values were checked using the R package coda (Plummer *et al.*, 2006) and outputs were analysed in the R package BAMMtools (Rabosky *et al.*, 2014). Second, we used BayesRate (Silvestro *et al.*, 2011) to test for different scenarios of diversification. We accounted for phylogenetic uncertainty by running the analysis over a sample of the posterior distribution of trees. Unlike BAMM, BayesRate does not estimate per-branch rate and require the specification of *a priori* branches with putatively varying rates of diversification. As we did not have strong assumptions about rate variation in the family, we implemented (1) a four-rate model in which each genus had its own rate and (2) a two-rate model with equal speciation and extinction rates for the sister genera *Cyathea* and *Alsophila* and a second set of rates for the remaining genera – *Gymnosphaera* and *Sphaeropteris*. We tested these two models against a constant-rate model by computing the marginal likelihoods of the three alternative scenarios. We then estimated speciation, extinction and net diversification rates under the best model by performing an MCMC analysis on a set of 100 trees randomly sampled from the posterior distribution of BEAST, running 10 million generations per tree. Finally, we computed the rates of net diversification (i.e. speciation minus extinction) per clade (for clade names see Supplementary Data Fig. S4) using the method of moments (Magallón and Sanderson, 2001; Meyer and Wiens, 2018). Net diversification rates were computed with both stem and crown ages and under zero extinction as well as high extinction (*ε* = 0.9; Magallón and Sanderson, 2001).

### Morphological data

We scored a matrix of three quantitative morphological traits (trunk height, lamina length and petiole length) for the species included in the phylogeny. All these variables relate to plant height and crown size, which vary with light availability (Arens and Sanchez Baracaldo, 2000). Published taxonomic revisions for the Neotropics (Windisch, 1977, 1978; Proctor, 1989; Rojas-Alvarado, 2001; Christenhusz, 2009; Lehnert, 2009, 2011, 2012, 2014, 2016; Lehnert and Weigand, 2013, 2017; Maciel, *et al.*, 2017), Africa (Holttum, 1981; Janssen and Rakotondrainibe, 2007, 2008) and Australasia (Holttum, 1959, 1963, 1964, 1965; Lehnert *et al.*, 2013) were consulted for character coding, which also drew from our own field observations. We computed overall body size, defined as the sum of trunk height, petiole length and lamina length. All values were then log-transformed. Body size has been shown by Ramírez-Barahona *et al.* (2016) to be one of the main drivers of Cyatheaceae diversification, although in their study low taxonomic sampling prevented any precise modelling of the evolution of this trait through time. We therefore investigated the dynamics of morphological change through time, by modelling body size evolution on our newly inferred time-calibrated phylogenetic tree under a relaxed Brownian motion process, using reversible-jump MCMC (Eastman *et al.*, 2011) as implemented in the function rjmcmc.bm of the R package geiger 2.0.6 (Harmon *et al.*, 2008). This Bayesian method, which is analogous to the phenotypic evolutionary rate analysis of BAMM, quantifies the heterogeneity in the rate of evolution of a continuous character across branches in the phylogeny by estimating the number and placement of rate shifts. We did not test multiple models of trait evolution but only focused on Brownian motion because our goal here was to investigate the variation in the rate of evolution of traits through time and between lineages. We performed an analysis of 10 million MCMC generations, sampling every 1000 generations. Convergence was checked using the R package coda (Plummer *et al.*, 2006). To make sure that phylogenetic uncertainty did not bias our estimates, we repeated the analysis on 100 trees randomly extracted from the posterior distribution of BEAST, each time running 5 million MCMC generations and sampling every 1000 generations. We then summarized the mean number of shifts and the mean rate per run. Finally, we re-evaluated the hypothesis that the rates of diversification in the Cyatheaceae were positively correlated with the rate of evolution of body size using structured rate permutations (STRAPP; Rabosky and Huang, 2016) as implemented in the R package BAMMtools (Rabosky *et al.*, 2014).

### Ecological data

We collected occurrence data for all species included in our phylogeny from nine online herbaria (E, GBIF, K, NY, SING, SpeciesLink, Tropicos, US, W) as well as three personal databases from our collaborators (Marcus Lehnert, Rodrigo Cámara Leret, Wilson D. Rodríguez Duque). All records were checked for (1) taxonomic correctness, using The Plant List (2013), and (2) regional GPS precision to country/state borders, using the World Administrative Borders from GADM (http://www.gadm.org/version2). We corrected the synonyms present in the databases with their accepted names and removed duplicates. Environmental data were extracted from CHELSA version 1.2 (Karger *et al.*, 2017), recalculated to 2 × 2 km resolution in R 3.4.3 (R Core Team, 2016). We used the extract function from the raster package 2.6–7 (Hijmans and van Etten, 2014) and interpolated values between the four nearest raster cells (method = bilinear) to account for local GPS errors at the scale of a few kilometres. For each occurrence, we extracted the values of annual precipitation and maximum temperature of the warmest month, i.e. the two climatic variables that have been shown to have the strongest limiting effect on tree fern distributions (Bystriakova *et al.*, 2011), and calculated their mean value for each species. The evolution of climatic preferences, along with morphological evolution, has been shown to be linked with increased diversification in the Cyatheaceae family (Ramírez-Barahona *et al.*, 2016). Thus, we analysed the two climatic variables following the same procedure as for body size, to re-assess the evolution of climatic preferences within the family and its impact on species diversification. We tested for heterogeneity in the rate of evolution of ecological traits by modelling the evolution of precipitation and temperature along the phylogenetic tree. We chose this single-variable approach because the use of principal component scores from multivariate analyses such as PCA or OMI can potentially biased the results of macroevolutionary analyses (Uyeda *et al.*, 2015). We performed a reversible-jump MCMC analysis of 10 million MCMC generations, sampling every 1000 generations, using the rjmcmc.bm function in R, and repeated the analysis on 100 phylogenetic trees from the posterior distribution. We then tested for trait-dependent diversification using STRAPP (Rabosky and Huang, 2016) as implemented in the R package BAMMtools (Rabosky *et al.*, 2014). Additionally, we recorded the mean elevation for the species included in the phylogeny using the same published taxonomic revisions that we consulted to score morphological data (see above) and visualized their elevational distribution by making the histogram of mean elevation and boxplots of the maximum elevation at different latitudes.

## RESULTS

### Phylogenetic reconstruction

**Fig. 1.**
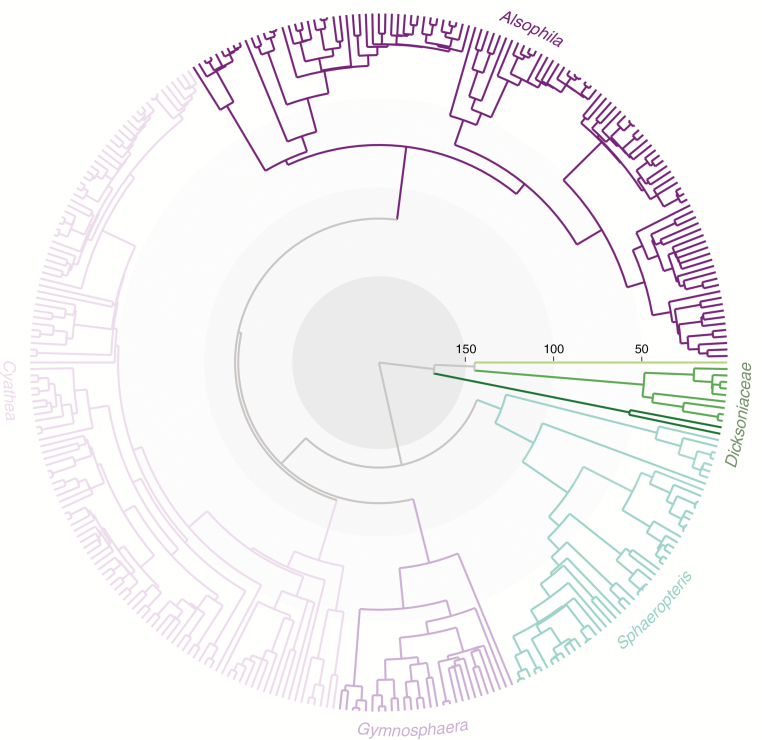
Time-calibrated phylogeny of the Cyatheaceae. Numbers indicate million years.

Our concatenated alignment of the three chloroplast genes was composed of 335 individuals and had a length of 3002 bp. For each DNA region, the best model of nucleotide substitution was GTR+G. The two runs of the FBD analysis in BEAST2 gave congruent results and all parameters had effective sample size values above 200. The topology of the maximum clade credibility tree supported the monophyly of the four genera (Fig. 1). The topology of the marginate-scaled clade was recovered with *Gymnosphaera* sister to a clade formed by *Alsophila* and *Cyathea*, but this relationship had a posterior probability of 0.33 and was therefore not supported (support values are provided in Supplementary Data Fig. S3). Relationships among closely related species were also poorly resolved, as indicated by low node posterior probabilities, especially within *Cyathea* and *Alsophila*. The Neotropical species of *Alsophila* formed a monophyletic clade. Divergence time estimates showed that Cyatheaceae diverged from their sister group, the Dicksoniaceae family, around 200 Ma [95 % HPD (highest posterior density) of stem node, 184.74–217.74] and the most recent common ancestor of all extant species is 140 Myr old (95 % HPD of crown node. 108.63–170.86). *Sphaeropteris* had a crown age of ~125 Myr and the remaining three genera had crown ages between 75 and 80 Myr. Overall, the node-dating analysis resulted in a similar topology with constantly younger age estimates, especially in the deeper nodes of the tree (Table 1).

**Table 1.** Clade age estimates from the node-dating (ND) and FBD analyses

	ND_min_	ND_max_	ND_mean_	FBD_min_	FBD_max_	FBD_mean_
Stem Cyatheaceae	147.48	172.40	157.75	184.74	217.74	199.47
Crown Cyatheaceae	101.06	136.83	117.80	108.63	170.86	139.42
Crown *Alsophila*	55.41	86.86	71.02	52.32	99.08	75.15
*A. abbottii*	25.41	47.56	40.04	18.32	42.81	30.08
*A. cuspidata*	15.63	35.05	26.01	18.48	47.31	34.60
*A. australis*	42.01	76.82	60.28	39.61	85.98	62.84
*A. humilis*	11.20	32.00	20.66	9.99	32.00	20.30
*A. manniana*	11.19	51.15	29.47	10.42	52.12	28.94
*A. smithii*	36.30	65.77	50.78	33.83	73.20	52.78
Crown *Cyathea*	57.56	85.42	71.45	60.34	105.78	81.30
*C. cnemidaria*	19.99	41.69	30.51	18.96	45.59	31.69
*C. armata*	36.85	64.63	50.46	39.26	77.19	57.70
*C. decurrens*	33.26	70.88	51.71	30.65	80.23	55.42
*C. divergens*	32.13	54.68	44.74	29.41	59.08	48.44
*C. gibbosa*	26.64	45.34	35.76	29.14	57.03	37.56
Crown *Gymnosphaera*	54.79	91.34	73.18	53.63	110.77	81.35
Gymnosphaera ‘Asian-clade’	33.29	60.83	46.51	31.95	68.28	48.79
Gymnosphaera ‘Malagasy-clade’	4.37	31.66	16.04	2.94	28.04	14.06
Crown *Sphaeropteris*	81.45	126.48	106.72	89.13	160.38	124.53
*S. horrida*	10.97	35.11	22.58	10.59	37.27	22.76
Sphaeropteris ‘Fourniera-clade’	16.27	56.28	35.18	15.34	60.81	35.35
*S. glauca*	29.08	56.79	49.33	27.97	62.17	50.82
Sphaeropteris ‘Sarcopholis-clade’	17.27	42.57	29.39	15.98	44.65	29.77
Sphaeropteris ‘Schizocaena-clade’	7.90	30.03	18.38	7.08	30.13	18.07
Crown Dicksoniaceae	117.83	151.95	136.93	128.38	201.51	167.10
Crown *Dicksonia*	40.01	72.26	55.03	23.76	72.24	47.46

### Diversification rates

**Fig. 2.**
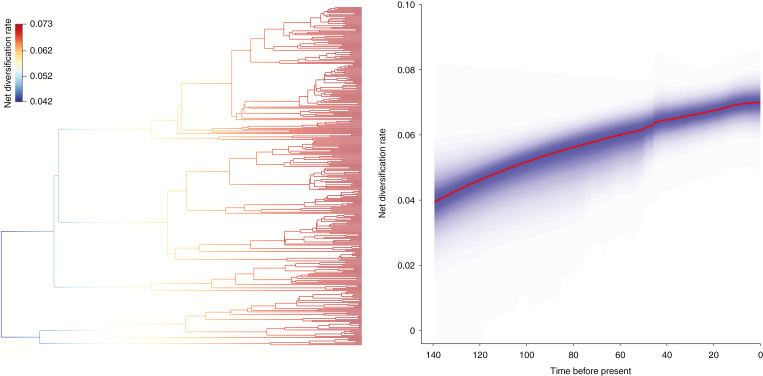
Results of BAMM analysis. Best configuration (left) and net diversification rate through time (right).

The results of the BAMM analyses with different priors on the number of rate shifts were similar, indicating that the analyses were robust to prior influence. Therefore, we present only the results of the analysis with a prior rate shift of 1. The rate of diversification slowly increased through time [mean net diversification rate 0.039 (95 % HPD 0.004–0.055) lineages per Myr at the root and 0.070 (95 % HPD 0.055–0.078) lineages per Myr at the tips] and BAMM detected no shift in the rate of diversification across the Cyatheaceae (Fig. 2). In BayesRate, although the model with the highest marginal likelihood was the two-rate model (Supplementary Data Table S2), with higher posterior net diversification rate for *Alsophila* and *Cyathea* (0.0608 lineages per Myr; 95 % HPD 0.040–0.084) than for *Gymnosphaera* and *Sphaeropteris* (0.0242 lineages per Myr; 95 % HPD 0.006–0.042), neither speciation nor extinction rates were actually significantly different in the two groups (Supplementary Data Fig. S7). Per-clade rates of net diversification estimated with the methods of moments varied between 0.025 and 0.142 lineages per Myr under no extinction and between 0.009 and 0.072 lineages per Myr when extinction was set to 0.9 (Supplementary Data Table S3). Despite some differences among rate estimates, all methods concurred in finding low diversification rates, with little evidence for strong rate heterogeneity across branches.

### Morphological evolution

The analysis of body size evolution showed a constant background rate of 0.0038 and a single shift with a posterior probability of 0.48 towards an increased rate detected in a small clade of four species of *Cyathea* (Supplementary Data Fig. S8). This result was robust to phylogenetic uncertainty, as shown by the distributions of the mean number of shifts and mean rate across the 100 additional runs (Supplementary Data Fig. S10). The result of the STRAPP analysis indicated no significant correlation between body size and net rates of diversification (Table 2).

**Table 2.** Correlation coefficients from STRAPP analyses

	Body size	Max temperature	Annual precipitation
*R*	−0.069	0.032	0.002
*P* value	0.904	0.940	0.928

### Climatic niche evolution

**Fig. 3.**
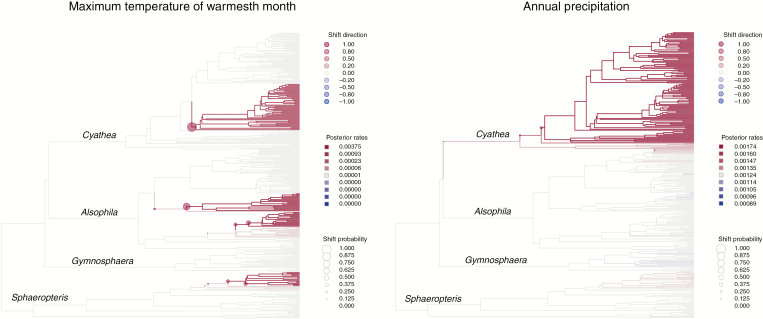
Posterior rates of the evolution of maximum temperature (left) and precipitation (right) modelled under a relaxed Brownian motion.

The analysis of trait evolution recovered a heterogeneous process for the evolution of temperature preferences, with a median posterior rate of 0.00001 and four shifts towards a rate of 0.00375 with posterior probabilities between 0.39 and 0.99 (Fig. 3), in the clades exhibiting the highest mean elevation and the widest elevational range. For annual precipitation, the median posterior rate was 0.00124 and rate acceleration was detected for the entire Neotropical *Cyathea* clade, which showed a rate of 0.00174 (Fig. 3). However, the posterior probability of this shift was only 0.14, indicating poor support (Fig. 3). The 100 replicated MCMC analyses showed that topological variation did not significantly impact the estimated rates and number of shifts of these two climatic variables (Supplementary Data Fig. S7). The STRAPP analysis showed that neither maximum temperature nor annual precipitation was correlated with net rates of diversification (Table 2).

## DISCUSSION

In this study, we evaluated several hypotheses related to the evolutionary history of a diverse clade of tree ferns and investigated the impact of morphological and climatic traits on the diversification of the group. We asked whether the build-up of species diversity in this group occurred through a steady accumulation of species through time or whether their great diversity originated instead from variations in the rate of diversification between lineages. We then investigated the role played by morphological and ecological traits in their evolution and tested the impact of these traits on the rates of diversification in the group.

Based on an unprecedented sampling of nearly half of extant species diversity and integrating data from the rich fossil record of tree ferns, we estimated divergence times using the FBD model. In agreement with published phylogenies (Janssen *et al.*, 2008; Korall and Pryer 2014; Ramírez-Barahona *et al.*, 2016), we found support for four main clades within Cyatheaceae, which are here referred to as the genera *Alsophila*, *Cyathea*, *Gymnosphaera* and *Sphaeropteris*. We also recovered a monophyletic clade for the Neotropical species of *Alsophila*, which were split between two different clades in a previous phylogeny with lower sampling (Korall and Pryer, 2014). As in most previous studies (Korall *et al.*, 2007; Janssen *et al.*, 2008; Korall and Pryer, 2014; Ramírez-Barahona *et al.*, 2016), we found low support for the phylogenetic relationships among *Alsophila*, *Cyathea* and *Gymnosphaera*, which is not surprising given that our molecular dataset is restricted to three chloroplast loci and that Cyatheaceae have a notably low rate of molecular evolution (Korall *et al.*, 2010). Since additional plastid sequences available in public repositories did not appear to improve substantially the resolution of the phylogeny, it is likely that only the addition of nuclear genes could yield a more robust resolution of the deep and shallow phylogenetic splits in this slowing evolving group. Alternatively, the low amount of divergence at the molecular level between *Alsophila*, *Cyathea* and *Gymnosphaera* (Fig. 1) may result from a high level of incomplete lineage sorting resulting in a ‘hard’ polytomy in this part of the phylogenetic tree.

Whether we used the FBD process or node dating, the recovered age estimates point to an older origin of the family than in most of the previous estimates (Janssen *et al.*, 2008; Korall and Pryer, 2014; Ramírez-Barahona *et al.*, 2016; Testo and Sundue, 2016; Lehtonen *et al.*, 2017). These differences in dating can be explained by several factors, including the different taxonomic assignment of the fossil *K. mirabilis* and the better taxon sampling and more realistic evolutionary models used in our phylogenetic analyses. The inclusion of old fossils representing stem lineages in the FBD analyses probably explains why the age estimates recovered by this method are older than those obtained with node calibration (Saladin *et al.*, 2017).

Results from our diversification analyses using different methods are coherent, and consistently indicate that the rate of diversification in Cyatheaceae was low and underwent very little variation throughout their long evolutionary history. This finding is consistent with a near-stable sampled diversity in the fossil record of Cyatheales (Lehtonen *et al.*, 2017), but contrasts strongly with previous reports of dramatic heterogeneity in the rates of diversification among Cyatheaceae (Janssen *et al.*, 2008; Ramírez-Barahona *et al.*, 2016). Previous studies were based on just about 10 % of the extant species and we believe that the 5-fold increase in taxon sampling in our analysis and advances in molecular dating methods are the basis of the divergent conclusions regarding the tempo of diversification in the group. Our estimates of the net diversification rates are 10–40 times slower than those of the fastest plant radiations reported to date, such as in *Dianthus* (Valente *et al.*, 2010), *Lupinus* (Hughes and Eastwood, 2006) or Andean Campanulaceae (Lagomarsino *et al.*, 2016). The estimates obtained for the Cyatheaceae are nevertheless among the intermediate values for fern families, where net diversification rates range from 0.001 in Thyrsopteridaceae to 0.132 in Athyriaceae (Testo and Sundue, 2018). However, apart from the Hymenophyllaceae, fern families with more than 600 species are generally younger clades that diversified at higher rates than the Cyatheaceae, whereas many of the early branching fern clades are characterized by much lower current diversity (Testo and Sundue, 2016, 2018). The Cyatheaceae therefore exhibit an uncommon pattern of an old lineage that has successfully diversified by accumulating species at a very low rate through time without any significant among-clade variation. The exact factors that have allowed the Cyatheaceae to diversify so extensively when compared with other tree fern families remain to be investigated, but this will require a much denser taxonomic sampling in the other families than what we have in our current study.

Our analyses have also shown that the modes of evolution differed substantially between morphological and climatic traits. The rate of body size evolution was remarkably low and constant across the phylogeny. This may seem surprising given that tree ferns differ considerably in size, ranging from minute rock-hugging species with leaves 15 cm long to very large species 20 m tall with 5-m long leaves. However, dwarf species are found only in Neotropical species of *Cyathea*, where dwarf growth has repeatedly evolved as an adaptation to exposed rocky outcrop habitats. This is most conspicuous in the *Hymenophyllopsis* group, with 11 species (Maciel *et al.*, 2017), but also concerns about 25 other species, which, based on morphology, should be distributed in several other clades. Due to the inaccessibility of these habitats, many of these species have only recently been described (Lehnert, 2006; Tejedor and Calatayud, 2017; Acuña-Tarazona *et al.*, 2018), and their diversity may be underestimated. Acceleration in the rate of body size evolution may thus be expected in dwarf *Cyathea*, but the shift detected in a clade of four of these species was not strongly supported (Supplementary Data Fig. S8), which may be due to the limited sampling of these groups. Although undersampling of dwarf species may underestimate morphological evolution in Neotropical *Cyathea*, it is unlikely to affect the overall result of low and nearly constant rate of body size evolution in the family. Korall *et al.* (2010) reported exceptionally slow rates of molecular evolution in tree ferns in comparison with other fern lineages, while a recent study found a negative correlation between substitution rates and body size in tree ferns (Barrera-Redondo *et al.*, 2018), but it is unclear whether this could actually be the underlying factor limiting the rate of phenotypic evolution. Given this result and the homogeneous rate of diversification estimated, it is thus not surprising that we did not detect any association between body size and diversification.

In contrast, the evolution of climatic preferences followed a more heterogeneous process. For precipitation, a small rate increase was detected in the clade of Neotropical *Cyathea*, whereas for temperature several shifts occurred independently within four clades in three different genera (Fig. 3). These four clades correspond to a primarily Andean clade in *Cyathea* and primarily New Guinean clades in *Alsophila* and *Sphaeropteris*. We interpret these patterns as the results of the topo-climatic heterogeneity of these mountains: the Andes and New Guinea are the most extensive and the highest tropical mountain systems, allowing niche shifts along the elevational gradient. However, whereas New Guinea mostly has humid climatic conditions in the mountains, the Andes have a much wider range of precipitation regimes, ranging from xeric to hyper-humid, which probably explains why only a single shift was detected for precipitation in the Neotropical clade of *Cyathea*. However, these shifts in the evolution of climatic preferences likely triggered by the colonization of mountain ranges were decoupled from species diversification, as shown by the lack of association between climatic traits and the rates of net diversification. This result was unexpected given that tropical mountains probably played an important role during the evolutionary history of Cyatheaceae. Indeed, these ecosystems harbour most of the global fern diversity, and colonization of montane habitats has been hypothesized to be the primary driver of diversification in several fern lineages (Kessler *et al.*, 2016). For example, in Polypodiaceae it has been shown that shifts in elevation, but not in leaf area, were positively associated with the rates of diversification, supporting the idea that exploration of new habitats rather than morphological innovation drove the diversification of the group (Sundue *et al.*, 2015). Environmental factors were also associated with diversification in a study using palaeontological data to investigate the drivers of diversification in all ferns (Lehtonen *et al.*, 2017), but the effect was correlated with extinction rates and not origination rates, which were largely diversity-dependent. In Cyatheaceae, tropical mountains likely provided new suitable habitats in which topographical complexity triggered allopatric speciation. Indeed, geographical isolation favoured by geological events has been suggested as an important factor in promoting differentiation in tree ferns (Ramírez-Barahona and Luna-Vega, 2015). Although the distribution of Cyatheaceae reaches high latitudes, their altitudinal range is greatest closer to the Equator and decreases towards higher latitudes (Supplementary Data Fig. S9). The appearance of montane habitats in tropical regions may have compensated the range contraction that Cyatheaceae underwent during their evolutionary history, as shown by the latitudinal range of their fossil record exceeding their extant distribution (Collinson, 2001). For example, Late Cretaceous fossil spores (*Kuylisporites*) similar to the spores of extant *Cyathea* are found in Siberia. Records of Cyatheaceae are also known from Antarctica in the Eocene, but are restricted to Central America by the Pliocene (Collinson, 2001). Therefore, rather than triggering a sharp biome shift that could increase net diversification rates, dispersals into higher elevations in the tropics may have simply represented an opportunity for Cyatheaceae to track or slightly expand their preferred climatic conditions. This is coherent with the fact that tree ferns are restricted to warm and humid environments with low seasonality and exhibit strong niche conservatism (Bystriakova *et al.*, 2011; Sosa *et al.*, 2016).

Our findings regarding morphological and climatic niche evolution disagree with the results of Ramírez-Barahona *et al.* (2016), who reported a positive correlation between the rates of body size and climatic niche evolution and the rates of diversification. Although our phylogeny is neither complete nor fully supported, we argue that our modelling of trait evolution, which took into account phylogenetic uncertainty, is more realistic than the approach used by Ramírez-Barahona *et al.* (2016), who circumvented a poor taxonomic sampling by modelling trait evolution on simulated trees, a procedure that has been shown to yield biased estimates (Rabosky, 2015). Conversely, and in line with our own results, a recent study across all ferns found a lack of association between the rates of diversification and the rates of body size evolution, a result that held true when looking at the Cyatheaceae in particular (Testo and Sundue, 2018).

### Conclusions

Our study provides a new perspective on the evolution of one of the most conspicuous vegetation elements of tropical montane forests. Rather than interpreting the present-day diversity of the Cyatheaceae as the result of adaptive bursts of diversification, as previously suggested (Ramírez-Barahona *et al.*, 2016), our findings indicate that their diversity steadily accumulated over time. Colonization of mountain habitats seems to have triggered a small acceleration of niche evolution but it was not followed by rapid radiation, unlike what has been observed in several plant groups, particularly in the Andes (Hughes and Atchison, 2015; Uribe-Convers and Tank, 2015; Pérez-Escobar *et al.*, 2017; Pouchon *et al.*, 2018; but see Wagner *et al.*, 2013; Salariato *et al.*, 2016). Many empirical examples support the view that changes in diversification rates linked to trait evolution are the primary driver of species diversity (Matuszak *et al.*, 2016; Onstein *et al.*, 2017), but counter-examples have shown that it is not a ubiquitous pattern (Vamosi and Vamosi, 2011; Rabosky *et al.*, 2012). The present study provides further evidence that a species-rich clade can result from the slow and steady accumulation of species over long evolutionary times and that rapid morphological differentiation, the evolution of key innovations and niche divergence are not a prerequisite for a clade to thrive for hundreds of millions of years.

## SUPPLEMENTARY DATA

Supplementary data are available online at https://academic.oup.com/aob and consist of the following. Figure S1: correlation of branch length of the two RaxML trees. Figure S2: boxplots of bootstrap values for the two phylogenetic trees. Figure S3: clades used to compute net diversification rates with the method of moments. Figure S4: maximum clade credibility tree from the BEAST2 analyses under the FBD process. Figure S5: comparison of parameter estimates from the FBD run with several fossils per taxon and a single fossil per taxon. Figure S6: correlation of clade age estimates between the FBD run with a single fossil per taxon and the FBD run with several fossils per taxon. Figure S7: differences between the posterior rates of the two groups for the best model in the BayesRates analysis. Figure S8: posterior rates of body size evolution modelled under relaxed Brownian motion. Figure S9: elevational distribution of the Cyatheaceae. Figure S10: histograms of the mean rate and mean number of shifts across the 100-replicate rjmcmc analysis for body size, annual precipitation and maximum temperature of warmest month. Table S1: fossils included in the FBD analysis. Table S2: model comparison in BayesRates and parameter estimation for the best model with two rates. Table S3: net diversification rate estimates from the method of moments.

mcz145_suppl_Supplementary_Data

## FUNDING

M.L. received funding from the German Research Foundation (LE 1826/3 and LE 1826/4) for conducting field work and sequencing, and from SYNTHESYS (GB-TAF-4927 and GB-TAF-6305) for herbarium studies. N.S. and M.K. received funding from the Swiss National Science Foundation (CRSII3-147630) and N.S. from the University of Lausanne.

## References

[CIT0001] Acuña-Tarazona M, Huamán-Melo E, Toledo-Aceves T, Mehltreter K 2018 *Cyathea leoniae* (Cyatheaceae), a new pinnate-pinnatifid tree fern species from Northern Peru. Phytotaxa 344: 191–197.

[CIT0002] Andriananjamanantsoa HN, Engberg S, Louis EE, Brouillet L 2016 Diversification of *Angraecum* (Orchidaceae, Vandeae) in Madagascar: revised phylogeny reveals species accumulation through time rather than rapid radiation. PLoS ONE 11: e0163194. 27669569 10.1371/journal.pone.0163194 PMC5036805

[CIT0003] Antonelli A, Zizka A, Silvestro D, Scharn R, Cascales-Miñana B, Bacon CD 2015 An engine for global plant diversity: highest evolutionary turnover and emigration in the American tropics. Frontiers in Genetics 6: 130. 25904934 10.3389/fgene.2015.00130 PMC4389561

[CIT0004] Arens NC, Sanchez Baracaldo P 2000 Variation in tree fern stipe length with canopy height: tracking preferred habitat through morphological change. American Fern Journal 90: 1–15.

[CIT0005] Baker WJ, Couvreur TLP 2013 Global biogeography and diversification of palms sheds light on the evolution of tropical lineages. II. Diversification history and origin of regional assemblages. Journal of Biogeography 40: 286–298.

[CIT0006] Barrera-Redondo J, Ramírez-Barahona S, Eguiarte LE 2018 Rates of molecular evolution in tree ferns are associated with body size, environmental temperature and biological productivity. Evolution 72: 1050–1062. 29604055 10.1111/evo.13475

[CIT0007] Bouckaert R, Heled J, Kühnert D, et al 2014 BEAST 2: a software platform for Bayesian evolutionary analysis. PLoS Computational Biology 10: e1003537. 24722319 10.1371/journal.pcbi.1003537 PMC3985171

[CIT0008] Breitkopf H, Onstein RE, Cafasso D, Schlüter PM, Cozzolino S 2015 Multiple shifts to different pollinators fuelled rapid diversification in sexually deceptive *Ophrys* orchids. New Phytologist 207: 377–389. 25521237 10.1111/nph.13219

[CIT0009] Brown JH 2014 Why are there so many species in the tropics? Journal of Biogeography 41: 8–22. 25684838 10.1111/jbi.12228 PMC4320694

[CIT0010] Bruun-Lund S, Verstraete B, Kjellberg F, Rønsted N 2018 Rush hour at the museum – diversification patterns provide new clues for the success of figs (*Ficus* L., Moraceae). Acta Oecologica 90: 4–11.

[CIT0011] Bystriakova N, Schneider H, Coomes D 2011 Evolution of the climatic niche in scaly tree ferns (Cyatheaceae, Polypodiopsida). Botanical Journal of the Linnean Society 165: 1–19.

[CIT0012] Cardillo M, Pratt R 2013 Evolution of a hotspot genus: geographic variation in speciation and extinction rates in *Banksia* (Proteaceae). BMC Evolutionary Biology 13: 155. 23957450 10.1186/1471-2148-13-155 PMC3751403

[CIT0013] Christenhusz MJM 2009 New combinations and an overview of *Cyathea* subg *Hymenophyllopsis* (Cyatheaceae). Phytotaxa 1: 37–42.

[CIT0014] Collinson ME 2001 Cainozoic ferns and their distribution. Brittonia 53: 173–235.

[CIT0015] Condamine FL, Silva-Brandão KL, Kergoat GJ, Sperling FAH 2012 Biogeographic and diversification patterns of Neotropical Troidini butterflies (Papilionidae) support a museum model of diversity dynamics for Amazonia. BMC Evolutionary Biology 12: 82. 22690927 10.1186/1471-2148-12-82 PMC3464124

[CIT0016] Couvreur TLP, Forest F, Baker WJ 2011 *a* Origin and global diversification patterns of tropical rain forests: inferences from a complete genus-level phylogeny of palms. BMC Biology 9: 44. 21679405 10.1186/1741-7007-9-44 PMC3142250

[CIT0017] Couvreur TLP, Pirie MD, Chatrou LW, et al 2011 *b* Early evolutionary history of the flowering plant family Annonaceae: steady diversification and boreotropical geodispersal. Journal of Biogeography 38: 664–680.

[CIT0018] Darriba D, Taboada GL, Doallo R, Posada D 2012 JModelTest 2: more models, new heuristics and parallel computing. Nature Methods 9: 772. 10.1038/nmeth.2109 PMC4594756 22847109

[CIT0103] Diaz LFH, Harmon LJ, Sugawara MT, Miller ET, Pennell MW 2019 Macroevolutionary diversification rates show time dependency. Proceedings of the National Academy of Sciences 116: 7403–7408. 10.1073/pnas.1818058116 PMC6462100 30910958

[CIT0019] Dong SY, Zuo ZY 2018 On the recognition of *Gymnosphaera* as a distinct genus in Cyatheaceae. Annals of the Missouri Botanical Garden 103: 1–24.

[CIT0021] Eastman JM, Alfaro ME, Joyce P, Hipp AL, Harmon LJ 2011 A novel comparative method for identifying shifts in the rate of character evolution on trees. Evolution 65: 3578–3589. 22133227 10.1111/j.1558-5646.2011.01401.x

[CIT0022] Eiserhardt WL, Couvreur TLP, Baker WJ 2017 Plant phylogeny as a window on the evolution of hyperdiversity in the tropical rainforest biome. New Phytologist 214: 1408–1422. 28277624 10.1111/nph.14516

[CIT0023] Harmon LJ, Weir JT, Brock CD, Glor RE, Challenger W 2008 GEIGER: investigating evolutionary radiations. Bioinformatics 24: 129–131. 18006550 10.1093/bioinformatics/btm538

[CIT0024] Heath TA, Hedtke SM, Hillis DM 2008 Taxon sampling and the accuracy of phylogenetic analyses. Journal of Systematics and Evolution 46: 239–257.

[CIT0025] Heath TA, Huelsenbeck JP, Stadler T 2014 The fossilized birth-death process for coherent calibration of divergence-time estimates. Proceedings of the National Academy of Sciences of the USA 111: E2957–E2966. 25009181 10.1073/pnas.1319091111 PMC4115571

[CIT0026] Henao Diaz LF, Harmon LJ, Sugawara MTC, Pennell MW 2019 Macroevolutionary diversification rates show time-dependency. bioRxiv. 10.1073/pnas.1818058116 PMC6462100 30910958

[CIT0027] Hijmans RJ 2015 Raster: geographic data analysis and modeling. R package version 2.4-15. http://CRAN.R-project.org/package=raster

[CIT0104] Hijmans RJ, van Etten J 2014 raster: Geographic data analysis and modeling. R package version 2.

[CIT0028] Holttum RE 1959 Pteridophyta. Flora Malesiana Series 2 1: 1–64.

[CIT0029] Holttum RE 1963 Cyatheaceae. Flora Malesiana Series 2 1: 65–176.

[CIT0030] Holttum RE 1964 The tree ferns of the genus *Cyathea* in Australasia and the Pacific. Blumea 12: 241–274.

[CIT0031] Holttum RE 1965 Tree ferns of the genus *Cyathea* Sm. in Asia (excluding Malaysia). Kew Bulletin 19: 463–487.

[CIT0032] Holttum RE 1981 The tree ferns of Africa. Kew Bulletin 36: 463–482.

[CIT0033] Hughes CE, Atchison GW 2015 The ubiquity of alpine plant radiations: from the Andes to the Hengduan Mountains. New Phytologist 207: 275–282. 25605002 10.1111/nph.13230

[CIT0034] Hughes C, Eastwood R 2006 Island radiation on a continental scale: exceptional rates of plant diversification after uplift of the Andes. Proceedings of the National Academy of Sciences of the USA 103: 10334–10339. 16801546 10.1073/pnas.0601928103 PMC1502458

[CIT0035] Janssen T, Rakotondrainibe F 2007 An update of the revision of *Cyathea* subgen. *Alsophila* sect. *Gymnosphaera* (Cyatheaceae) in Madagascar and the Comoros including a discussion of putative hybridization events. Adansonia 29: 195–213.

[CIT0036] Janssen T, Rakotondrainibe F 2008 A revision of the indusiate scaly tree ferns (Cyatheaceae, *Cyathea* subgen. *Alsophila* sect. *Alsophila*) in Madagascar, the Comoros and the Seychelles. Adansonia 30: 221–376.

[CIT0037] Janssen T, Bystriakova N, Rakotondrainibe F, Coomes D, Labat J-N, Schneider H 2008 Neoendemism in Madagascan scaly tree ferns results from recent, coincident diversification bursts. Evolution 62: 1876–1889. 18452578 10.1111/j.1558-5646.2008.00408.x

[CIT0038] Karger DN, Conrad O, Böhner J, et al 2017 Climatologies at high resolution for the earth’s land surface areas. Scientific Data 4: 170122. 28872642 10.1038/sdata.2017.122 PMC5584396

[CIT0039] Katoh K, Misawa K, Kuma K, Miyata T 2002 MAFFT: a novel method for rapid multiple sequence alignment based on fast Fourier transform. Nucleic Acids Research 30: 3059–3066. 12136088 10.1093/nar/gkf436 PMC135756

[CIT0040] Kessler M, Karger DN, Kluge J 2016 Elevational diversity patterns as an example for evolutionary and ecological dynamics in ferns and lycophytes. Journal of Systematics and Evolution 54: 617–625.

[CIT0041] Korall P, Pryer KM 2014 Global biogeography of scaly tree ferns (Cyatheaceae): evidence for Gondwanan vicariance and limited transoceanic dispersal. Journal of Biogeography 41: 402–413. 25435648 10.1111/jbi.12222 PMC4238398

[CIT0042] Korall P, Conant DS, Metzgar JS, Schneider H, Pryer KM 2007 A molecular phylogeny of scaly tree ferns (Cyatheaceae). American Journal of Botany 94: 873–886. 21636456 10.3732/ajb.94.5.873

[CIT0043] Korall P, Schuettpelz E, Pryer KM 2010 Abrupt deceleration of molecular evolution linked to the origin of arborescence in ferns. Evolution 64: 2786–2792. 20394660 10.1111/j.1558-5646.2010.01000.x

[CIT0044] Lagomarsino LP, Condamine FL, Antonelli A, Mulch A, Davis CC 2016 The abiotic and biotic drivers of rapid diversification in Andean bellflowers (Campanulaceae). New Phytologist 210: 1430–1442. 26990796 10.1111/nph.13920 PMC4950005

[CIT0045] Lehnert M 2006 New species and records of tree ferns (Cyatheaceae, Pteridophyta) from the northern Andes. Organisms Diversity and Evolution 6: 321–322.

[CIT0046] Lehnert M 2009 Resolving the *Cyathea caracasana* complex (Polypodiopsida: Cyatheaceae). Stuttgarter Beiträge zur Naturkunde A, Neue Serie 2: 409–445.

[CIT0047] Lehnert M 2011 Species of *Cyathea* in America related to the western Pacific species *C. decurrens*. Phytotaxa 26: 39–59.

[CIT0048] Lehnert M 2012 A synopsis of the species of *Cyathea* (Cyatheaceae-Polypodiopsida) with pinnate to pinnate-pinnatifid frond. Phytotaxa 61: 17–36.

[CIT0049] Lehnert M 2014 Do you know *Cyathea divergens* (Cyatheaceae-Polypodiopsida)? Phytotaxa 161: 1–42.

[CIT0050] Lehnert M 2016 A synopsis of the exindusiate species of *Cyathea* (Cyatheaceae-Polypodiopsida) with bipinnate-pinnatifid or more complex fronds, with a revision of the *C. lasiosora* complex. Phytotaxa 243: 1–53.

[CIT0051] Lehnert M, Weigand A 2013 A proposal to distinguish several taxa in the Brazilian tree fern *Cyathea corcovadensis* (Cyatheaceae). Phytotaxa 155: 35–49.

[CIT0052] Lehnert M, Weigand A 2017 A synopsis of the Neotropical species of *Cyathea* (Cyatheaceae; Polypodiopsida) with bipinnate fronds. Brittonia 69: 71–90.

[CIT0053] Lehnert M, Coritico FP, Darnaedi D, et al 2013 Taxonomic and ecological notes on the *Alsophila hornei* complex (Cyatheaceae-Polypodiopsida), with the description of the new species *A. phlebodes* from New Guinea. Systematic Botany 38: 875–886.

[CIT0054] Lehtonen S, Silvestro D, Karger DN, et al 2017 Environmentally driven extinction and opportunistic origination explain fern diversification patterns. Scientific Reports 7: 4831. 28684788 10.1038/s41598-017-05263-7 PMC5500532

[CIT0055] Litsios G, Sims CA, Wüest RO, Pearman PB, Zimmermann NE, Salamin N 2012 Mutualism with sea anemones triggered the adaptive radiation of clownfishes. BMC Evolutionary Biology 12: 212. 23122007 10.1186/1471-2148-12-212 PMC3532366

[CIT0056] Maciel S, Lehnert M, Hirai RY, Prado J 2017 Three new species of the *Cyathea* “*Hymenophyllopsis*” clade (Cyatheaceae) from Venezuela and Brazil. Phytotaxa 329: 159–166.

[CIT0057] Magallón S, Sanderson MJ 2001 Absolute diversification rates in angiosperm clades. Evolution 55: 1762–1780. 11681732 10.1111/j.0014-3820.2001.tb00826.x

[CIT0058] Matuszak S, Favre A, Schnitzler J, Muellner-Riehl AN 2016 Key innovations and climatic niche divergence as drivers of diversification in subtropical Gentianinae in southeastern and eastern Asia. American Journal of Botany 103: 899–911. 27208358 10.3732/ajb.1500352

[CIT0059] Meyer ALS, Wiens JJ 2018 Estimating diversification rates for higher taxa: BAMM can give problematic estimates of rates and rate shifts. Evolution 72: 39–53. 29055133 10.1111/evo.13378

[CIT0060] Mohr BAR, Lazarus DB 1994 Paleobiogeographic distribution of *Kuylisporites* and its possible relationship to the extant fern genus *Cnemidaria* (Cyatheaceae). Annals of the Missouri Botanical Garden 81: 758–767.

[CIT0061] Moore BR, Höhna S, May MR, Rannala B, Huelsenbeck JP 2016 Critically evaluating the theory and performance of Bayesian analysis of macroevolutionary mixtures. Proceedings of the National Academy of Sciences of the USA 113: 9569–9574. 27512038 10.1073/pnas.1518659113 PMC5003228

[CIT0062] Nagalingum NS, Schneider H, Pryer KM 2007 Molecular phylogenetic relationships and morphological evolution in the heterosporous fern genus *Marsilea*. Systematic Botany 32: 16–25.

[CIT0063] Noben S, Kessler M, Quandt D, et al 2017 Biogeography of the Gondwanan tree fern family Dicksoniaceae – a tale of vicariance, dispersal and extinction. Journal of Biogeography 44: 2648–2659.

[CIT0064] Onstein RE, Baker WJ, Couvreur TLP, Faurby S, Svenning JC, Kissling WD 2017 Frugivory-related traits promote speciation of tropical palms. Nature Ecology and Evolution 1: 1903–1911. 29062122 10.1038/s41559-017-0348-7

[CIT0065] Pérez-Escobar OA, Chomicki G, Condamine FL, et al 2017 Recent origin and rapid speciation of Neotropical orchids in the world’s richest plant biodiversity hotspot. New Phytologist 215: 891–905. 28631324 10.1111/nph.14629 PMC5575461

[CIT0066] Plummer M, Best N, Cowles K, Vines K 2006 CODA: convergence diagnosis and output analysis for MCMC. R News 6: 7–11.

[CIT0067] Pouchon C, Fernández A, Nassar JM, et al 2018 Phylogenomic analysis of the explosive adaptive radiation of the *Espeletia* complex (Asteraceae) in the tropical Andes. Systematic Biology 67: 1041–1060. 30339252 10.1093/sysbio/syy022

[CIT0068] PPG I 2016 A community-derived classification for extant lycophytes and ferns. Journal of Systematics and Evolution 54: 563–603.

[CIT0069] Proctor GR 1989 Ferns of Puerto Rico and the Virgin Islands. Memoirs of the New York Botanical Garden, Vol. 53. New York: New York Botanical Garden Press.

[CIT0105] R Core Team. 2016 R: A language and environment for statistical computing. Versión 3.4.3. Vienna, Austria: R Foundation for Statistical Computing.

[CIT0070] Rabosky DL 2014 Automatic detection of key innovations, rate shifts, and diversity-dependence on phylogenetic trees. PLoS ONE 9: e89543. 24586858 10.1371/journal.pone.0089543 PMC3935878

[CIT0071] Rabosky DL 2015 No substitute for real data: a cautionary note on the use of phylogenies from birth-death polytomy resolvers for downstream comparative analyses. Evolution 69: 3207–3216. 26552857 10.1111/evo.12817

[CIT0072] Rabosky DL, Huang H 2016 A robust semi-parametric test for detecting trait-dependent diversification. Systematic Biology 65: 181–193. 26396091 10.1093/sysbio/syv066

[CIT0073] Rabosky DL, Slater GJ, Alfaro ME 2012 Clade age and species richness are decoupled across the eukaryotic tree of life. PLoS Biology 10: e1001381. 22969411 10.1371/journal.pbio.1001381 PMC3433737

[CIT0074] Rabosky DL, Grundler M, Anderson C, et al. 2014 BAMMtools: an R package for the analysis of evolutionary dynamics on phylogenetic trees. Methods in Ecology and Evolution 5: 701–707.

[CIT0075] Rabosky DL, Mitchell JS, Chang J 2017 Is BAMM flawed? Theoretical and practical concerns in the analysis of multi-rate diversification models. Systematic Biology 66: 477–498. 28334223 10.1093/sysbio/syx037 PMC5790138

[CIT0076] Rambaut A, Drummond AJ 2009 Tracer: MCMC trace analysis tool, version 1.5. http://tree.bio.ed.ac.uk/software/tracer.

[CIT0077] Ramírez-Barahona S, Luna-Vega I 2015 Geographic differentiation of tree ferns (Cyatheales) in tropical America. American Fern Journal 105: 73–85.

[CIT0078] Ramírez-Barahona S, Barrera-Redondo J, Eguiarte LE 2016 Rates of ecological divergence and body size evolution are correlated with species diversification in scaly tree ferns. Proceedings of the Royal Society B: Biological Sciences 283: 20161098. 10.1098/rspb.2016.1098 PMC4947896 27412279

[CIT0079] Rojas-Alvarado AF 2001 Nuevas especies, nombres nuevamente utilizados y nuevas distribuciones en los helechos arborescentes (Filicales: Cyatheaceae) para el Neotrópico. Revista de Biología Tropical 49: 453–466. 11935896

[CIT0081] Saladin B, Leslie AB, Wüest RO, et al 2017 Fossils matter: improved estimates of divergence times in *Pinus* reveal older diversification. BMC Evolutionary Biology 17: 95. 28376717 10.1186/s12862-017-0941-z PMC5381128

[CIT0082] Salariato DL, Zuloaga FO, Franzke A, Mummenhoff K, Al-Shehbaz IA 2016 Diversification patterns in the CES clade (Brassicaceae tribes Cremolobeae, Eudemeae, Schizopetaleae) in Andean South America. Botanical Journal of the Linnean Society 181: 543–566.

[CIT0083] Serrano-Serrano ML, Rolland J, Clark JL, Salamin N, Perret M 2017 Hummingbird pollination and the diversification of angiosperms: an old and successful association in Gesneriaceae. Proceedings of the Royal Society B: Biological Sciences 284: 20162816. 10.1098/rspb.2016.2816 PMC5394660 28381621

[CIT0084] Silvestro D, Schnitzler J, Zizka G 2011 A Bayesian framework to estimate diversification rates and their variation through time and space. BMC Evolutionary Biology 11: 311. 22013891 10.1186/1471-2148-11-311 PMC3224121

[CIT0085] Silvestro D, Zizka G, Schulte K 2014 Disentangling the effects of key innovations on the diversification of Bromelioideae (Bromeliaceae). Evolution 68: 163–175. 24372602 10.1111/evo.12236

[CIT0086] Sosa V, Ornelas JF, Ramírez-Barahona S, Gándara E 2016 Historical reconstruction of climatic and elevation preferences and the evolution of cloud forest-adapted tree ferns in Mesoamerica. PeerJ 4: e2696. 27896030 10.7717/peerj.2696 PMC5119233

[CIT0087] Sundue MA, Testo WL, Ranker TA 2015 Morphological innovation, ecological opportunity, and the radiation of a major vascular epiphyte lineage. Evolution 69: 2482–2495. 26257202 10.1111/evo.12749

[CIT0088] Tejedor A, Calatayud G 2017 Eleven new scaly tree ferns (*Cyathea*: Cyatheaceae) from Peru. American Fern Journal 107: 156–191.

[CIT0089] Testo W, Sundue M 2016 A 4000-species dataset provides new insight into the evolution of ferns. Molecular Phylogenetics and Evolution 105: 200–211. 27621129 10.1016/j.ympev.2016.09.003

[CIT0090] Testo WL, Sundue MA 2018 Are rates of species diversification and body size evolution coupled in the ferns? American Journal of Botany 105: 525–535. 29637539 10.1002/ajb2.1044

[CIT0091] The Plant List 2013 http://www.theplantlist.org/.

[CIT0092] Uribe-Convers S, Tank DC 2015 Shifts in diversification rates linked to biogeographic movement into new areas: an example of a recent radiation in the Andes. American Journal of Botany 102: 1854–1869. 26542843 10.3732/ajb.1500229

[CIT0093] Uyeda JC, Caetano DS, Pennell MW 2015 Comparative analysis of principal components can be misleading. Systematic Biology 64: 677–689. 25841167 10.1093/sysbio/syv019

[CIT0094] Vaidya G, Lohman DJ, Meier R 2011 SequenceMatrix: concatenation software for the fast assembly of multi-gene datasets with character set and codon information. Cladistics 27: 171–180. 10.1111/j.1096-0031.2010.00329.x 34875773

[CIT0095] Valente LM, Savolainen V, Vargas P 2010 Unparalleled rates of species diversification in Europe. Proceedings of the Royal Society B: Biological Sciences 277: 1489–1496. 10.1098/rspb.2009.2163 PMC2871840 20106850

[CIT0096] Vamosi JC, Vamosi SM 2011 Factors influencing diversification in angiosperms: at the crossroads of intrinsic and extrinsic traits. American Journal of Botany 98: 460–471. 21613139 10.3732/ajb.1000311

[CIT0097] de Vos JM, Hughes CE, Schneeweiss GM, Moore BR, Conti E 2014 Heterostyly accelerates diversification via reduced extinction in primroses. Proceedings of the Royal Society B: Biological Sciences 281: 20140075. 10.1098/rspb.2014.0075 PMC4043087 24759859

[CIT0098] Wagner N, Silvestro D, Brie D, et al 2013 Spatio-temporal evolution of *Fosterella* (Bromeliaceae) in the Central Andean biodiversity hotspot. Journal of Biogeography 40: 869–880.

[CIT0099] Wiens JJ 2017 What explains patterns of biodiversity across the Tree of Life? New research is revealing the causes of the dramatic variation in species numbers across branches of the Tree of Life. BioEssays 39: 1600128. 10.1002/bies.201600128 28054372

[CIT0100] Wilson R, Heinrichs J, Hentschel J, Gradstein SR, Schneider H 2007 Steady diversification of derived liverworts under Tertiary climatic fluctuations. Biology Letters 3: 566–569. 17686755 10.1098/rsbl.2007.0287 PMC2391190

[CIT0101] Windisch PG 1977 Synopsis of the genus *Sphaeropteris* with a revision of the neotropical exindusiate species. Botanische Jahrbücher der Systematik 92: 176–198.

[CIT0102] Windisch PG 1978 The systematics of the group of *Sphaeropteris hirsuta* (Cyatheaceae). In: Maguire B, ed. The botany of the Guayana Highland. Memoirs of the New York Botanical Garden, Vol. 29 New York: New York Botanical Garden Press, 2–22.

