# Supplementary material for: Slowly but surely: gradual diversification and phenotypic evolution in the hyper-diverse tree fern family Cyatheaceae
Source: Ann Bot. 2019 Sep 28;125(1):93–103. doi: 10.1093/aob/mcz145 (PMC6948215; doi:10.1093/aob/mcz145)
Supplement: mcz145_suppl_Supplementary_Data [file mcz145_suppl_supplementary_data.docx]

**Supplementary Data**

**Loiseau et al. – Slowly but surely: gradual diversification and phenotypic evolution in the hyper-diverse tree fern family Cyatheaceae**

**Phylogenetic reconstruction with additional Genbank data**

We retrieved sequence data from Genbank of the four additionnal markers for which at least 20% of the species in our dataset were available: atpB-rbcL (790bp, 81 species), rbcL (106 species, 1,428bp), rbcL-accD (1,597 bp, 78 species, SQD1 (532 bp, 70 species). Then, we performed phylogenetic reconstruction with RAxML on A) our original alignment and B) our alignment combined with Genbank data, using a GTR-GAMMA model of substitution and the -autoMRE option to perform bootstrap replicates. We also computed normalized and weighted Robinson-fould distances between the two phylogenetic trees and compared their bootstrap values using boxplots. Next, to be able to compare the branch lengths obtained with the two datasets, we used the combined alignment with Genbank data to perform branch length estimation on the fixed topology given by our phylogenetic tree, using the -f e option in RAxML. We then estimated the correlation between the set of branch lengths estimated with the extended alignment and the set of branch lengths of our phylogenetic tree with linear regression.

**Results**

Robinson-Foulds distances: normalized RF = 0.364; weighted RF= 0.102


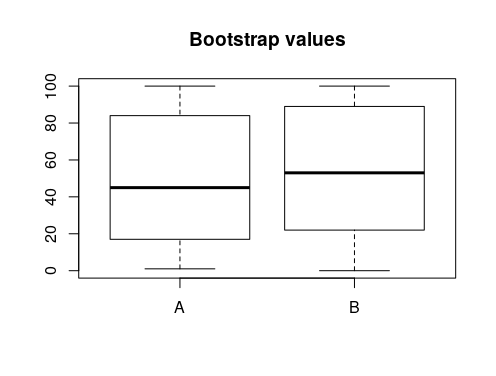

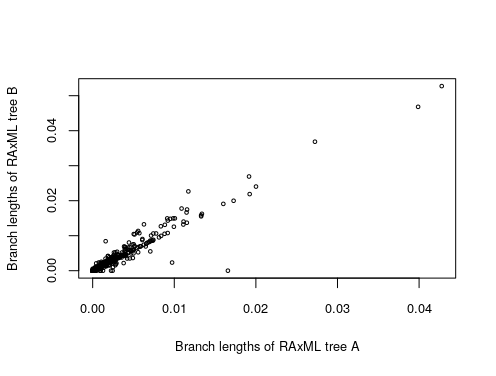


| **Supplementary Figure 1.** Correlation of branch length of the two RAxML trees (R^2^=0,93, *p-value* = 2.2e-16). |  | **Supplementary Figure 2.** Boxplots of bootstrap values for the two phylogenetic trees. |
| --- | --- | --- |


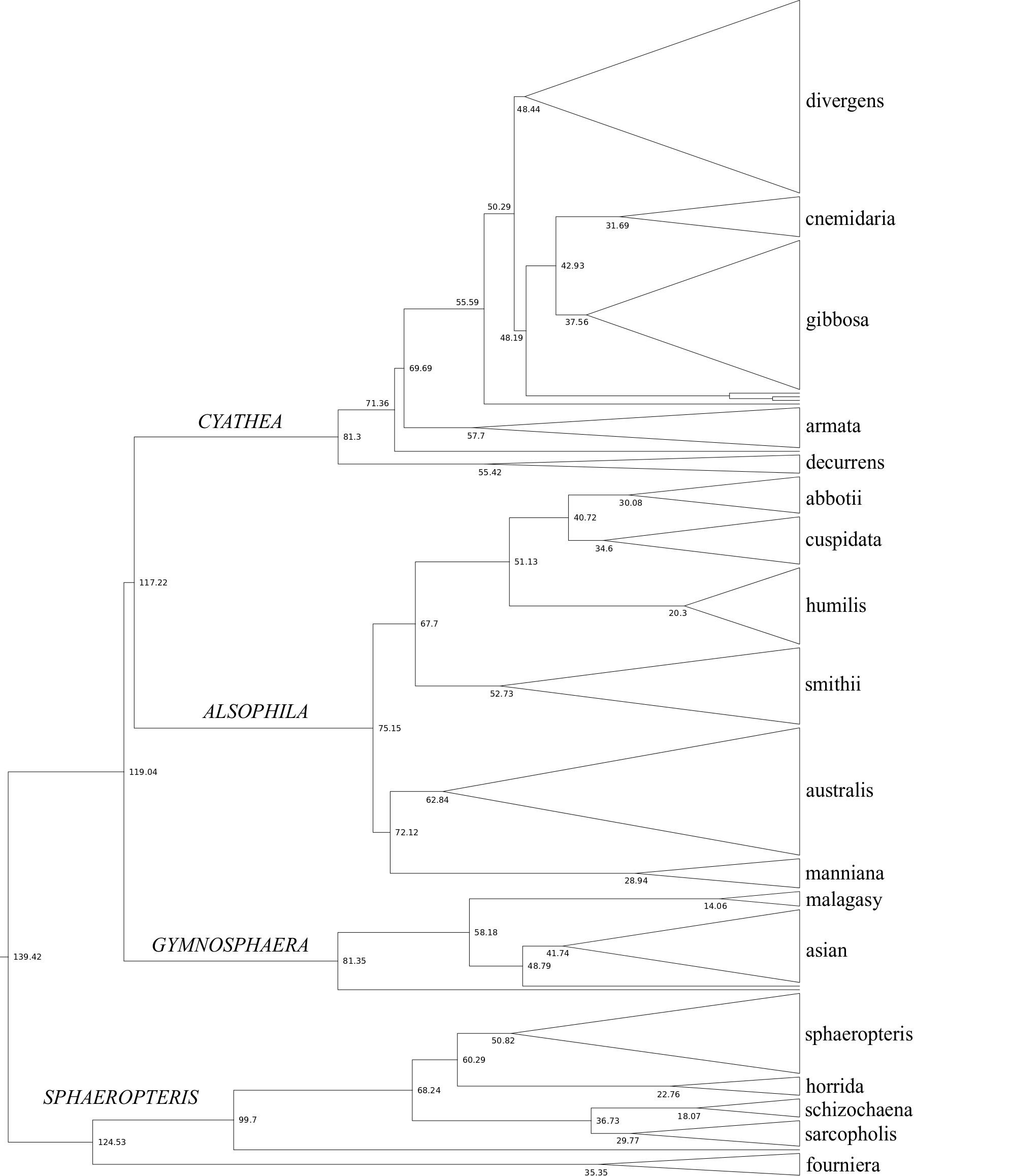


**Supplementary Figure 3.** Clades used to compute net diversification rates with the methods of moments.

**Supplementary Figure 4 (next 2 pages).** Maximum clade credibility tree from the BEAST2 analyses under the FBD process. Node numbers indicate posterior probabilities and node bars show the 95% credibility interval of node age estimates.

**
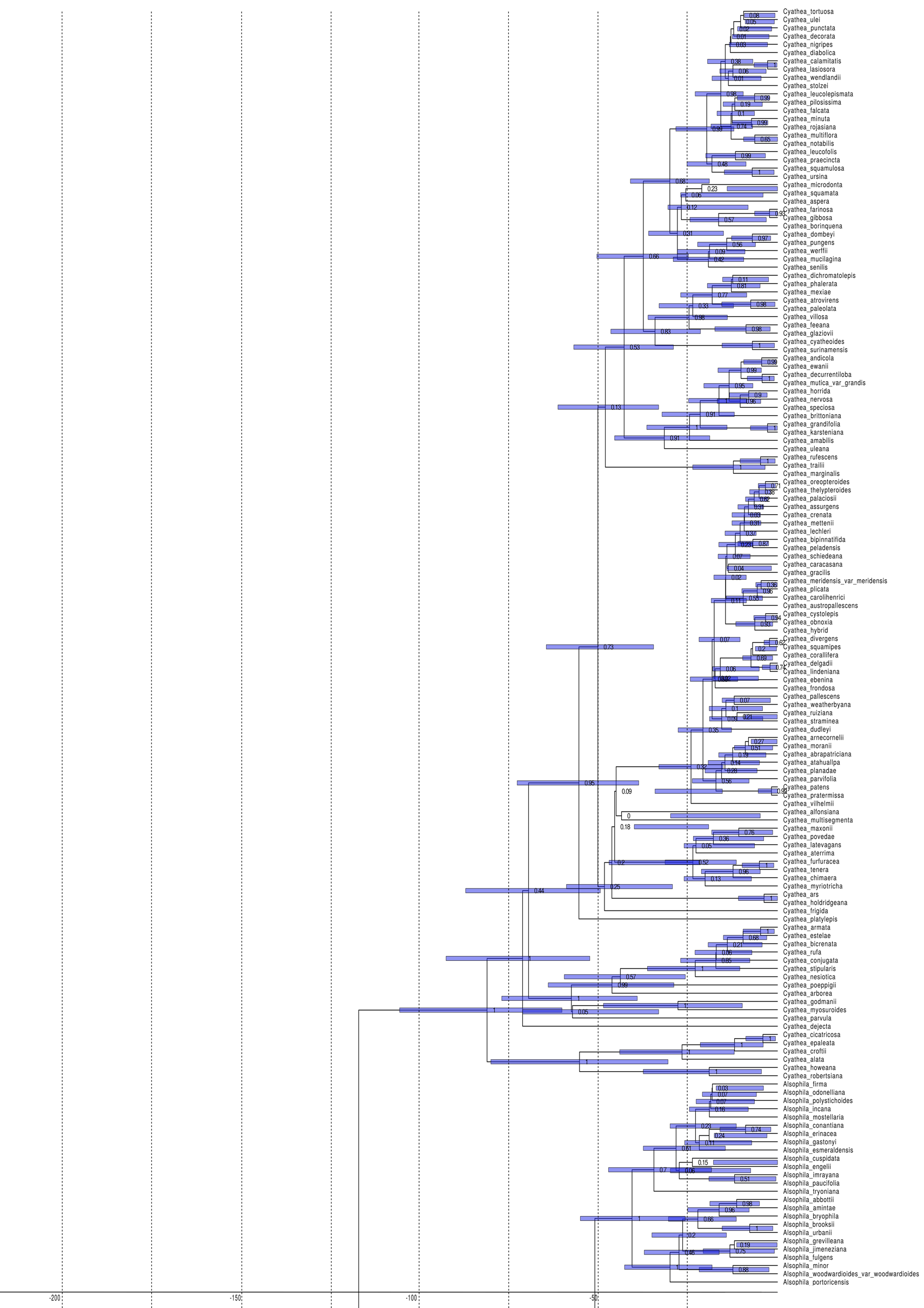
**

**
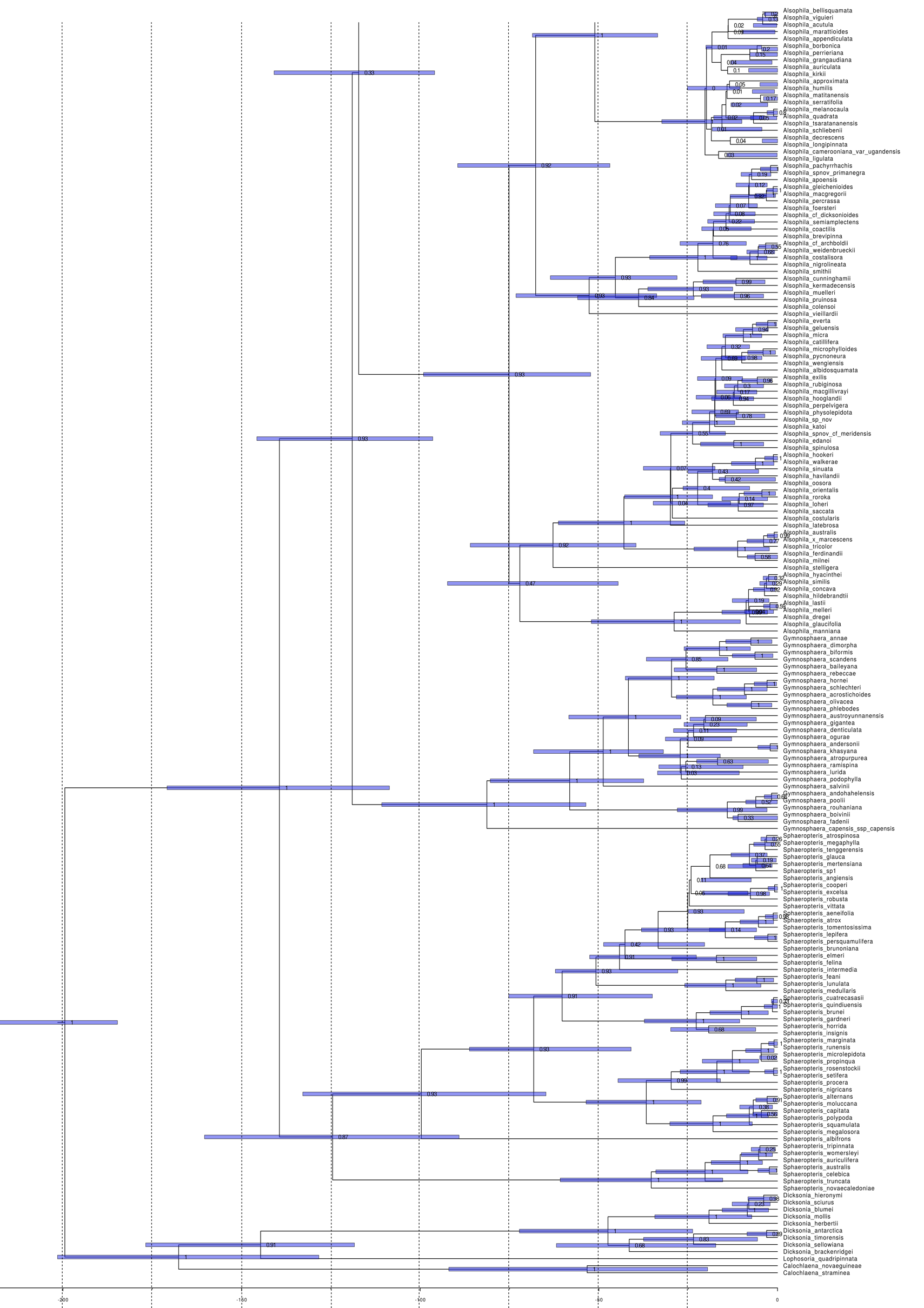
**

**Divergence time estimation with node calibration**

Divergence time estimation using node calibration was performed in BEAST 2.4.7, on the same molecular dataset as for the Fossilized Birth-Death analysis. We used a GTR+G model of substitution for each partition, a birth-death tree prior and an uncorrelated relaxed clock. We performed two runs of 200 million generations, sampling every 20,000 generations. The two runs were combined using Logcombiner and the maximum clade credibility tree obtained with TreeAnnotator, after checking for convergence in Tracer 1.5.0. We implemented the following four calibration points:

1) The first appearance of the fossil genus *Cyathocaulis* (Upper Jurassic: 145-165.5 Myr ago) was used to constrained the root node of our tree, i.e. the stem node of Cyatheaceae, as it has been shown to be a stem lineage of Cyatheaceae, based on a morphological phylogenetic reconstruction combining extant and extinct tree ferns (Lantz et al. 1999).

2) The earliest *Kuylisporites* fossil spores, described as *K.mirabilis,* from the Cenomanian of Siberia (93.9-100.5 Myr ago) were used to constrain the stem node of *Cyathea*, as the morphology of *Kuylisporites* fossil spores is found only in extant species of *Cyathea.* (Mohr and Lazarus 1994)*⁠*

3) The fossil *Dicksonia antartica* from the Eocene of King George's island (33.9-56 Myr ago) was used to constrain the crown node of the Australo-American clade of *Dicksonia* (Noben et al. 2017).

4) The macrofossil record of fertile frond from the Aptian (113-125 Myr ago) of King George's island, described as *Lophosoria cupulatus,* was used to constrain the stem node of *Lophosoria*. (Hill et al. 2001)⁠.

For all calibration points we applied a lognormal prior distribution, starting at the fossil's minimum age and with the 95% interval reaching the fossil's maximum age.

**Divergence time estimation with single fossil occurrence**

The fossil data had several occurrences for each fossil. We tested whether the use of all fossil occurrences gave different results in the FBD analyses than when randomly selecting a single of the occurrences per fossil. The two different procedures do not significantly impact the estimation of the parameters of the FBD process. Although keeping a single occurrence has an effect on the rate and turnover of the FBD, diversification rate is not impacted (see Sup. Mat. Fig. 5). Keeping only one fossil per taxa resulted in slightly older estimates and wider confidence intervals, however we show that the age estimates from the two FBD runs are very strongly correlated (Sup. Mat. Fig. 6). Therefore, we believe that the approach we took, following the practice done in the literature in which multiple fossils of the same taxa are used (e.g. Eguchi et al. 2016, Renner et al. 2016), does not bias the estimation of diversification rates.

**
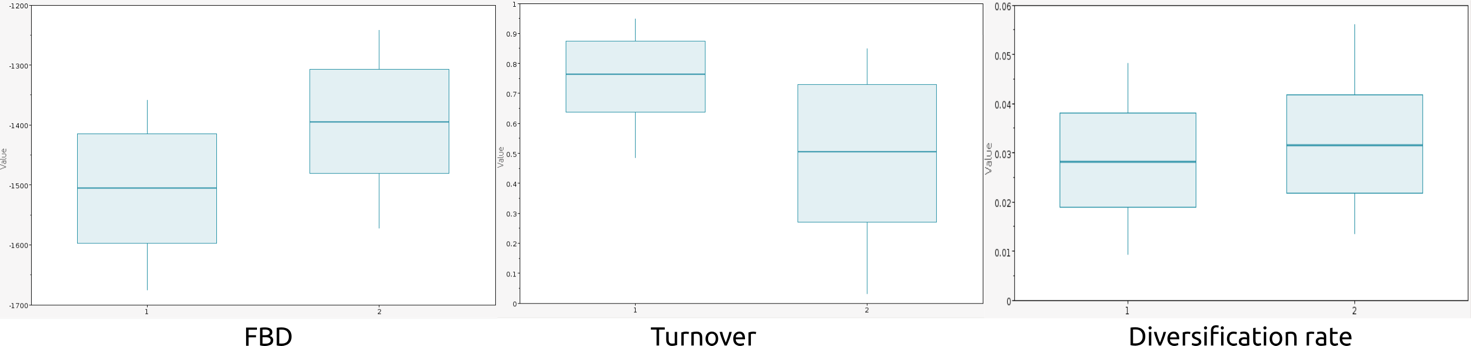
**

**Supplementary Figure 5.** Comparison of parameter estimates from the FBD run with several fossil per taxon (left boxplot) and a single fossil per taxon (right boxplot).


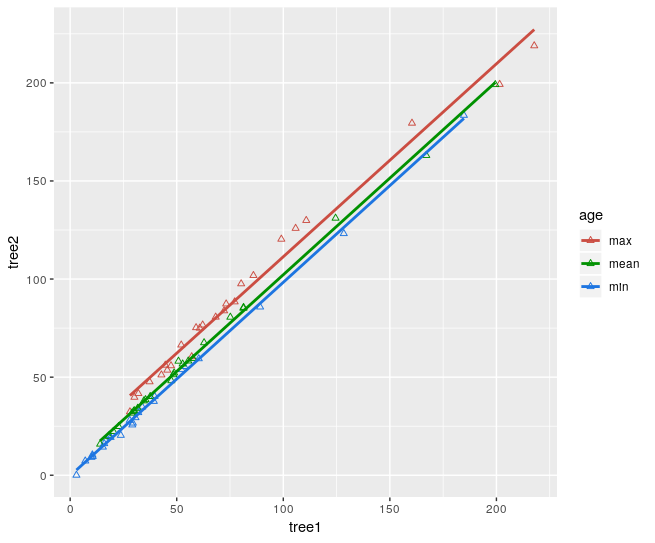
**Supplementary Figure 6.** Correlation of clade age estimates between the FBD run with a single fossil per taxon (y axis) and the FBD run with several fossil per taxon (x axis).


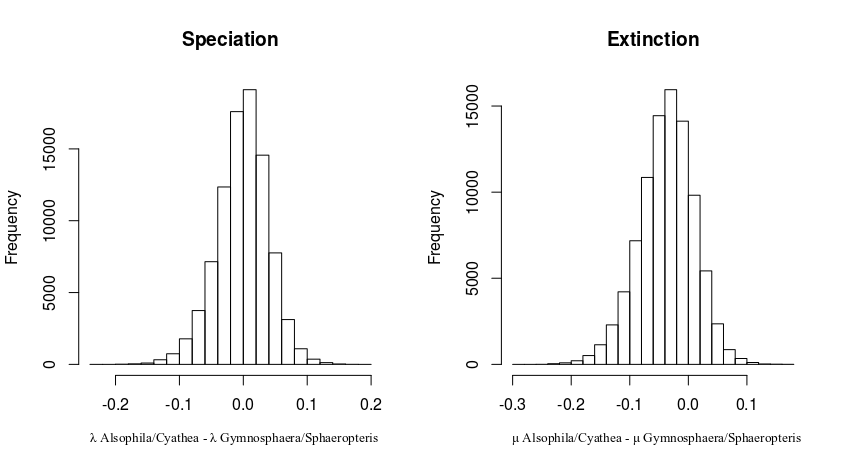


**Supplementary Figure 7.** Differences between the posterior rates of the two groups for the best model in the Bayesrates analysis. Distributions show the posterior rates of the *Alsophila/Cyathea* group minus the posterior rates of the *Gymnosphaera/Sphaeropteris* group for speciation (left) and extinction rates (right). The 95% HPD of the two distributions include zero, indicating that the difference between the two clades is not significant.


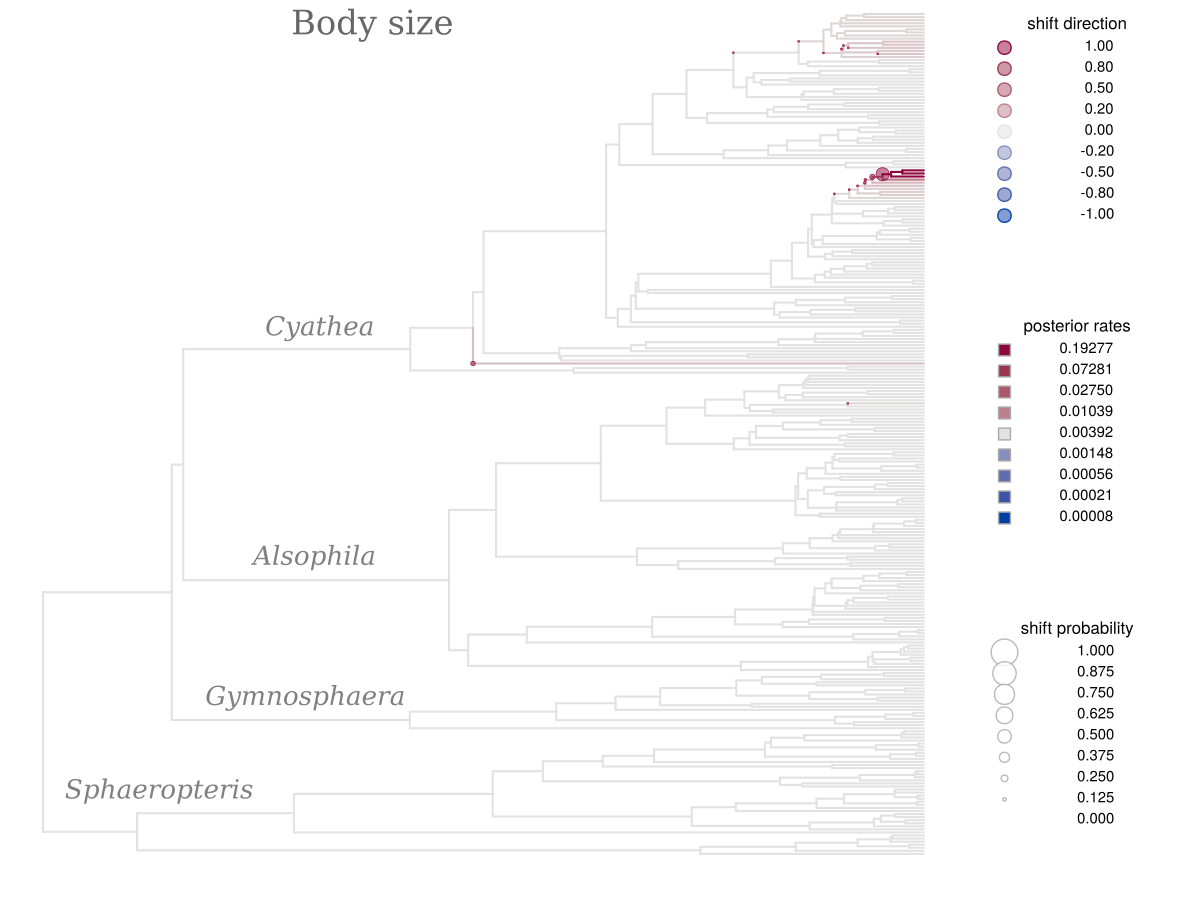


**Supplementary Figure 8.** Posterior rates of body size evolution modelled under relaxed Brownian motion.


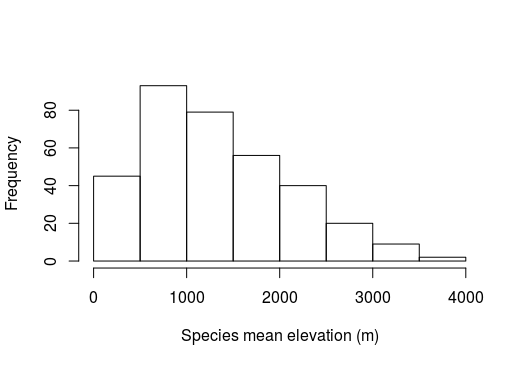

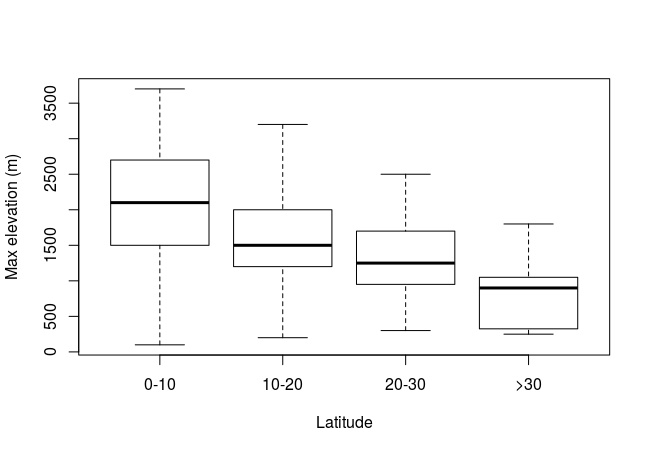


**Supplementary Figure 9.** Altitudinal distribution of the Cyatheaceae. Left: Histogram of species mean elevation. Right: Boxplots of maximum elevation per species per category of latitude.

**
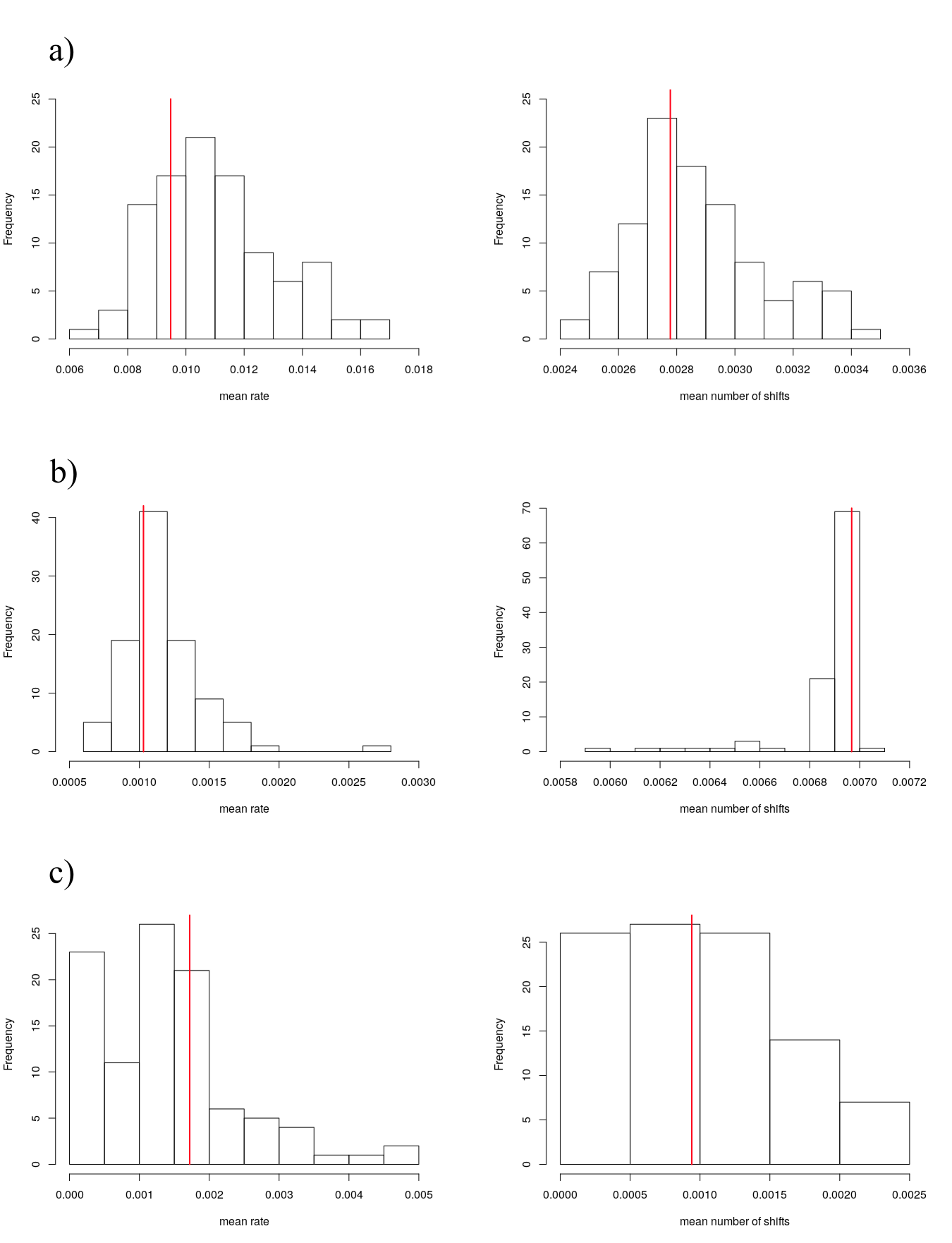
**

**Supplementary Figure 10.** Histograms of the mean rate and mean number of shifts across the 100 replicates rjmcmc analysis for body size (a), annual precipitation (b) and maximum temperature of warmest month (c). Red lines show the mean value from the rjmcmc analysis performed on the mcc tree.

**Supplementary Table 1.** Fossils included in the Fossilized Birth-Death analysis.

**Supplementary Table 2.** Model comparison in BayesRates (upper) and parameter estimation for the best model with two rates (lower).

**Supplementary Table 3.** Net diversification rate estimates from the methods of moments. The location of each clade in the phylogenetic tree of Cyatheaceae is indicated on the figure below.

**References**

Eguchi, S., and M.N. Tamura. 2016. Evolutionary timescale of monocots determined by the fossilized birth‐death model using a large number of fossil records. Evolution. 70:1136–1144.

Hill, R. S., M. K. Macphail, and G. J. Jordan. 2001. Macrofossils associated with the fossil fern spore *Cyatheacidites annulatus* and their significance for Southern hemisphere biogeography. Rev. Palaeobot. Palynol. 116:195–202.

Lantz, T., G. Rothwell, and R. Stockey. 1999. *Conantiopteris schuchmanii*, gen. et sp. nov., and the role of fossils in resolving the phylogeny of Cyatheaceae s.l. J. Plant Res. 112:361–381.

Mohr, B. A. R., and D. B. Lazarus. 1994. Paleobiogeographic distribution of *Kuylisporites* and its possible relationship to the extant fern genus *Cnemidaria* (Cyatheaceae). Ann. Missouri Bot. Gard. 81:758–767.

Noben, S., M. Kessler, D. Quandt, A. Weigand, S. Wicke, M. Krug, and M. Lehnert. 2017. Biogeography of the Gondwanan tree fern family Dicksoniaceae — A tale of vicariance, dispersal and extinction. J. Biogeo. 44:2648–2659.

Renner, S.S., G.W. Grimm, P. Kapli, and T. Denk. 2016. Species relationships and divergence times in beeches: new insights from the inclusion of 53 young and old fossils in a birth–death clock model. Phil. Trans. R. Soc. B. 371.
